# A Precise Visual Method for Narrow Butt Detection in Specular Reflection Workpiece Welding

**DOI:** 10.3390/s16091480

**Published:** 2016-09-13

**Authors:** Jinle Zeng, Baohua Chang, Dong Du, Yuxiang Hong, Shuhe Chang, Yirong Zou

**Affiliations:** Key Laboratory for Advanced Materials Processing Technology, Ministry of Education, Department of Mechanial Engineering, Tsinghua University, Beijing 100084, China; zengjl12@mails.tsinghua.edu.cn (J.Z.); bhchang@tsinghua.edu.cn (B.C.); hongyuxiang@tsinghua.edu.cn (Y.H.); changsh15@mails.tsinghua.edu.cn (S.C.); zouyr@tsinghua-tj.org (Y.Z.)

**Keywords:** welding process control, seam tracking, specular reflection workpiece welding, narrow butt, visual detection

## Abstract

During the complex path workpiece welding, it is important to keep the welding torch aligned with the groove center using a visual seam detection method, so that the deviation between the torch and the groove can be corrected automatically. However, when detecting the narrow butt of a specular reflection workpiece, the existing methods may fail because of the extremely small groove width and the poor imaging quality. This paper proposes a novel detection method to solve these issues. We design a uniform surface light source to get high signal-to-noise ratio images against the specular reflection effect, and a double-line laser light source is used to obtain the workpiece surface equation relative to the torch. Two light sources are switched on alternately and the camera is synchronized to capture images when each light is on; then the position and pose between the torch and the groove can be obtained nearly at the same time. Experimental results show that our method can detect the groove effectively and efficiently during the welding process. The image resolution is 12.5 μm and the processing time is less than 10 ms per frame. This indicates our method can be applied to real-time narrow butt detection during high-speed welding process.

## 1. Introduction

During complex path welding, the welding torch must be aligned with the groove center to achieve good welding quality. It is necessary to control the motion path of the torch or the workpiece in real time during the whole welding process. Currently, the “teaching-playback” and offline programming techniques are widely used in robotic welding. The welding path is recorded beforehand and then the robots can move along the recorded path during welding. However, in the actual welding conditions, the machining errors, assembly errors and thermal deformation will lead to a deviation between the actual and the recorded welding paths. Therefore, it is critical to develop automatic seam detection technologies to recognize the position of the actual welding path. Thus, the deviation between the torch and the workpiece can be corrected automatically [1].

The visual detection method is the most promising seam recognition technology, due to its abundant information, non-contact with workpiece, high precision and good sensitivity [2,3,4]. However, there are serious challenges when detecting the seam of the aerospace components [5,6,7]:

1. Specular reflection surface of the workpiece. The components are usually made of aluminum alloy, titanium alloy and stainless steel, which may strongly reflect the incident light. For example, the reflectance of aluminum to visible light can be over 95% [8]. The strong specular reflection effect will lead to the non-uniform grayscale distribution in the images, lowering the signal-to-noise ratio (SNR) of imaging.
2. Small width of the narrow butt joint. The workpieces to be welded are mounted close to each other, which is called the “narrow butt”. The width of the butt joint is significantly small (smaller than 0.2 mm), which requires a precise detection method.
3. Small welding region. The components are usually joined by gas tungsten arc welding (GTAW), friction stir welding or high energy beam welding (such as laser, electron beam and plasma arc welding) methods. The welding region is usually small, so that the welding quality would greatly deteriorate even though there is a small deviation between the torch and the welding path.
4. High welding speed. The welding speed can reach 3 m/min or higher in high energy beam welding, which requires a real-time detection method. It is essential to get high SNR images and develop efficient image processing algorithm in order to meet the real-time detection needs.

**Figure 1:**
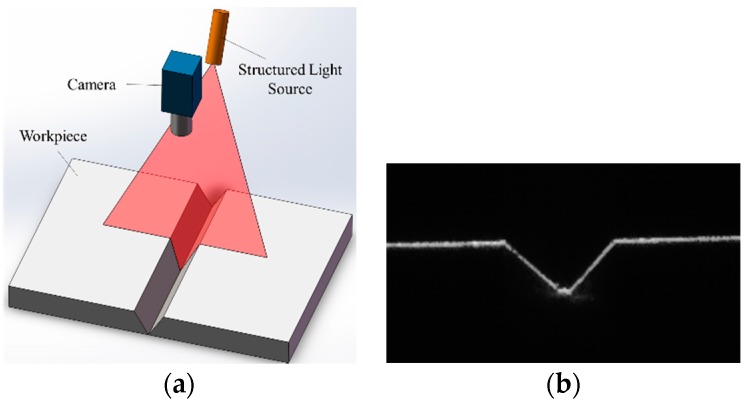
The structured light detection method used in industry. (**a**) The structured light detection method applied to V-groove detection; (**b**) The V-groove image captured by the camera.

**Figure 2:**
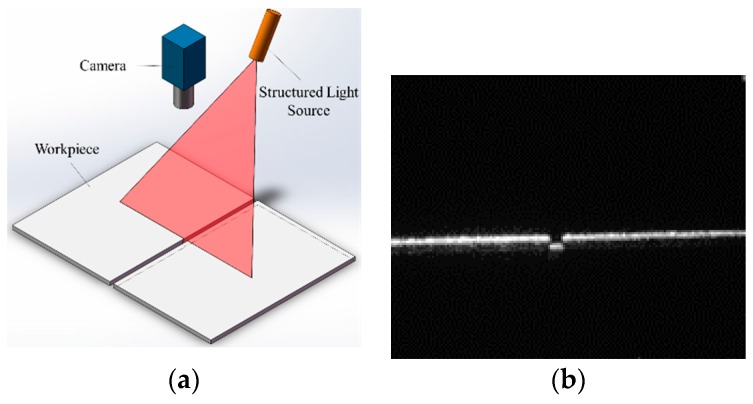

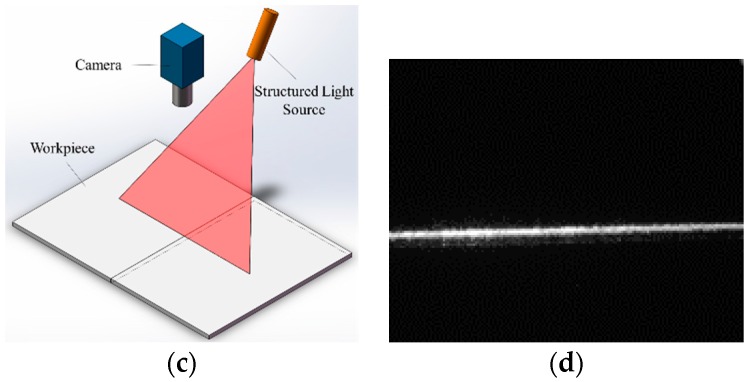
Structured light detection method applied to narrow butt detection. (**a**) Structured light is projected onto the groove with 1 mm width; (**b**) Captured image when the groove width is 1 mm; (**c**) Structured light is projected onto the groove with less than 0.2 mm width; (**d**) Captured image when the groove width is less than 0.2 mm.

Nowadays, the structured light detection method is the most widely used in the industry [9,10,11]. There have been lots of commercial structured light sensors developed by Meta Vision, Servo Robot, etc. [12,13,14]. In this method, a laser stripe is projected onto the groove and its distortion mirrors the geometric differences between the groove and the base metal [15,16,17,18,19,20,21]. This method is suitable when there are significant geometric differences between the groove and the base metal as shown in Figure 1. However, as shown in Figure 2, when detecting narrow gaps such as 0.2 mm, the laser line distortion may not be significant enough. Moreover, the width of the laser stripe would become thick due to the specular reflection effects, lowering the detection accuracy. Therefore, the structured light method is not applicable in narrow butt detection.

To overcome the existing problems in structured light detection methods, researchers have proposed many other visual methods to achieve narrow butt detection. Huang projected a line laser stripe onto the workpiece surface and captured the images of the laser speckles [22]. The discontinuity of the speckles indicates the region of the groove. Zheng projected a circular laser spot onto the workpiece surface [23]. The continuous dark curve in the image indicates the position of the groove and at the same time, the position and pose between the torch and the workpiece can be calculated according to the dimensional change and shape distortion of the spot. Chen, Ma, and Shen proposed several novel narrow butt detection methods [24,25,26]. The differences between the groove and the base metal are highlighted in arc or LED lighting conditions.

However, although some active lighting methods are proposed in these studies, there is still a lack of solutions for the specular reflection effect; the signal-to-noise ratio of the images captured in the previous researches is pretty low [22,23,24,25,26]. The grayscale distribution of the laser speckles or the workpiece surface is not uniform [24,25,26]; There are several highlighted regions caused by specular reflection effects while the grayscales in other regions are lower [23]. The grayscale differences between the groove and the base metal are not so distinct and the groove region is significantly polluted by noise. The poor imaging quality might affect the subsequent image processing algorithm, which is not helpful for an accurate and real-time detection of the narrow butt joint. Furthermore, most of the existing methods can only obtain the transversal offset of the groove, but the information of 3D offsets and the pose between the torch and the workpiece is very important in complex path welding. Therefore, these existing methods might not be applicable to narrow butt detection in specular workpiece welding.

In this paper, we propose a precise and effective visual narrow butt detection method in specular reflection workpiece welding. We will study the in-depth visual sensing method of specular reflection surface and propose a detection method to obtain the 3D offsets and pose between the torch and the workpiece. First, based on the reflection characteristics of the workpiece, a uniform surface light source was designed to construct the uniform lighting condition, in order to get high SNR images against the adverse effects of specular reflection. The grayscale differences between the groove and the base metal were greatly highlighted, which was beneficial to an accurate detection of the groove. Then, a double-line laser stripe was projected onto the workpiece to obtain the equation of the workpiece surface in the camera coordinate system. Considering the transformation matrix between the camera and torch coordinate systems, the position and pose of the workpiece relative to the torch were calculated. Afterwards, a visual detection sensor was developed and a “shutter-synchronized dual light stroboscopic” trigger method was used to obtain two kinds of information nearly simultaneously when each light source is on. Finally, welding experiments were carried out to verify the applicability of our method.

The rest of the paper is organized as follows: Section 2 will illustrate the principles of our visual sensing method against specular reflection effects. The corresponding image processing algorithm will be proposed to detect the pixel coordinates of the narrow butt. Section 3 will introduce a method to detect the position and pose between the torch and the workpiece. A double-line laser stripe is used to detect the workpiece equation relative to the welding torch. Combining the research results above, we will develop a visual sensor of narrow butt detection in Section 4. In Section 5, welding experiments were conducted and the sensor was used to detect the narrow butt in real time. Finally, conclusions are given in Section 6.

## 2. Visual Detection Method of Narrow Butt of Specular Reflection Workpiece

### 2.1. Principles of the Narrow Butt Visual Sensing Method of Specular Reflection Workpieces

**Figure 3:**
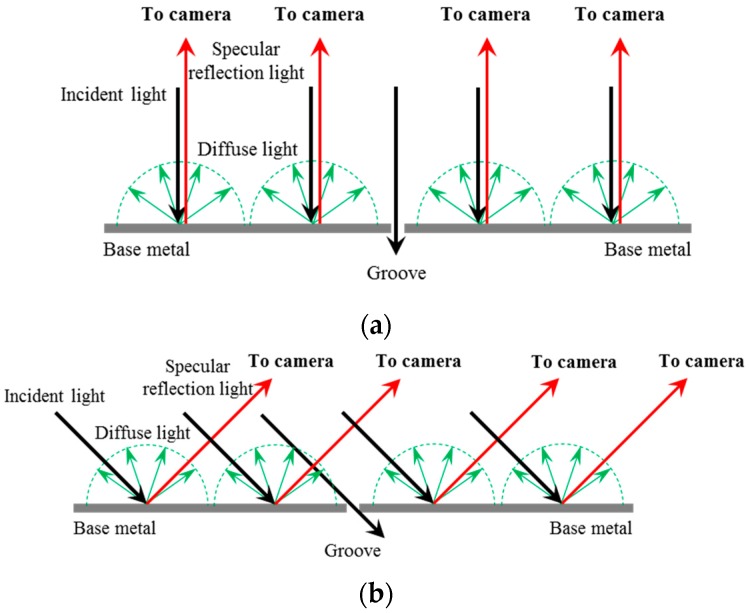
The reflection characteristic differences between the groove and the base metal. (**a**) The incident light is normal to the surface of the base metal; (**b**) The incident light is not normal to the surface of the base metal.

Figure 3 shows the reflection characteristic differences between the groove and the base metal regions. On one hand, when the uniform light is projected onto the base metal, the incident light is reflected and almost returns along the specular reflection axis because of the strong specular reflection effect. The light intensity near the specular reflection axis is much greater than other directions. On the other hand, the light projected onto the groove cannot return due to the gap of the narrow butt joint. Therefore, the intensity of the reflected light in the groove region is relatively lower.

As a result, the proposed visual sensing method must meet the following needs to achieve an effective detection: first, a uniform lighting condition must be constructed and thus we can obtain the images with uniform grayscale distribution, overcoming the adverse effects of specular reflection; and second, the camera must be mounted near the specular reflection axis, by which the grayscale differences between the groove and the base metal can be highlighted to the utmost extent.

**Figure 4:**
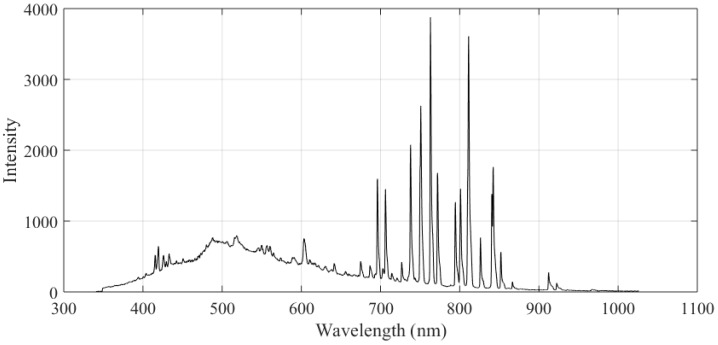
The spectrum of the arc light during aluminum alloy welding using GTAW method.

Figure 4 shows the typical spectrum of the arc light during aluminum alloy welding using the GTAW method. The spectrum was obtained by a USB2000 spectrometer. The intensity of the arc light is relatively low in the range of 610 nm to 690 nm. In order to eliminate the interference from the arc, the wavelength of the active light source we will design should be within this range.

**Figure 5:**
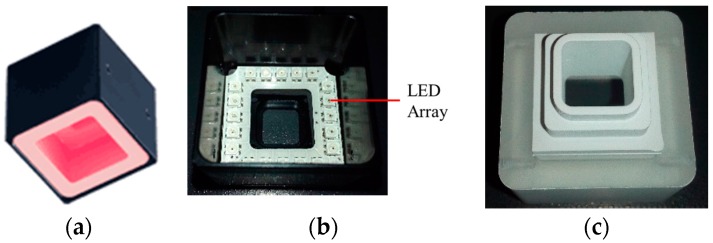

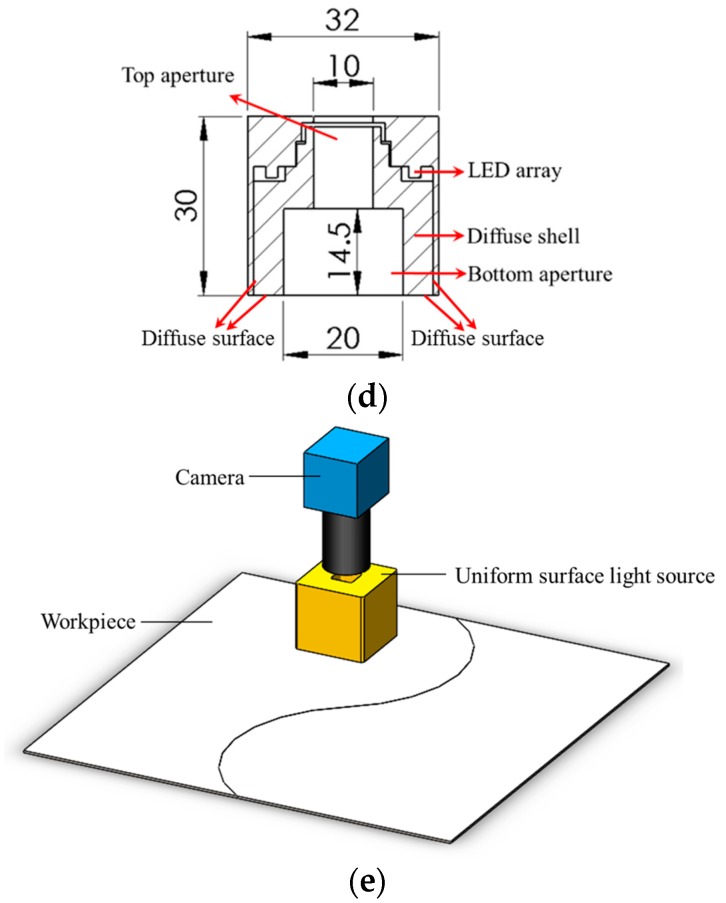
The designed uniform surface light source and its installation. (**a**) The uniform surface light source; (**b**) The outermost shell and the LED array of the light source; (**c**) The diffuse shell of the light source; (**d**) The inner structure of the light source, including the diffuse surface, aperture, and LED array; (**e**) The relative position of the camera, the uniform surface light source, and the workpiece.

In this paper, we design a uniform surface light source (USLS) by analytic optical simulation and verify its lighting effect by experiments. The designed USLS is shown in Figure 5 of which the central wavelength is 630 nm and maximum power is 6 W. It is composed of an LED array with 20 LEDs, a diffuse shell, a top aperture, and a bottom aperture. The dimensions of the top and bottom apertures are 10 mm × 10 mm and 20 mm × 20 mm, respectively. 

**Figure 6:**
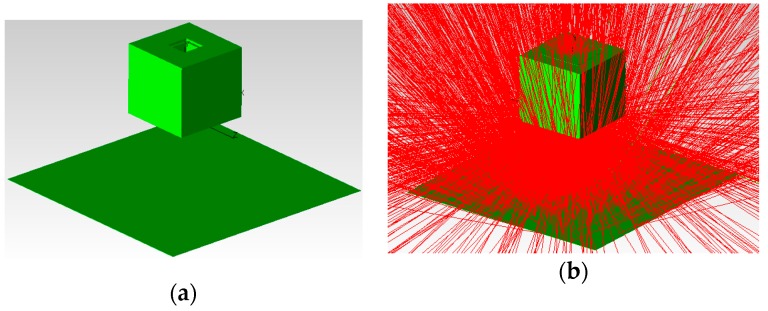
The simulation process of the uniform surface light source using Tracepro. (**a**) The simulation scenario; (**b**) The result after ray tracing.

**Figure 7:**
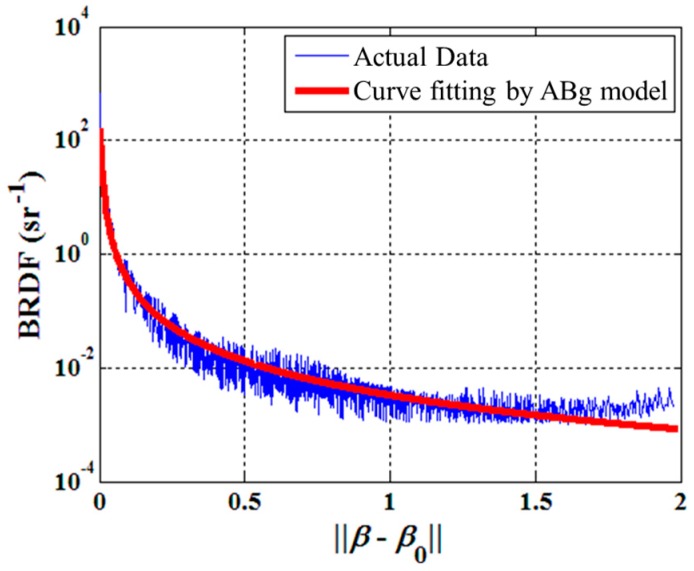
The fitted ABg model of aluminum.

The diffuse shell is designed to reflect the light emitted from the LED array to construct a uniform lighting condition. The bottom and side surface of the diffuse shell are covered with high reflection coatings. The light emitted from the LED array is reflected many times by the coated surface and then projected onto the workpiece surface; then the reflected light from the workpiece surface travels through the bottom and top aperture in succession and is finally captured by the camera as shown in Figure 5e. In order to evaluate the illumination effect of the USLS, we used Tracepro to calculate the luminous flux distribution emitted from the top aperture as shown in Figure 6. Tracepro is an optical simulation software, which can calculate the luminous flux distribution on the surfaces using ray tracing algorithm. The distance between the USLS and the workpiece surface was set to be 30 mm, 40 mm, and 50 mm, respectively. Then we calculated the luminous flux distribution in a 16 mm × 16 mm region which is 0.5 mm above the top aperture. The Bidirectional Reflection Distribution Function (BRDF) data of aluminum from MERL BRDF database [8] is applied as the reflection characteristic of the workpiece during the simulation process. These data are fitted by the ABg BRDF model beforehand as shown in Figure 7, which is regarded as an input parameter in the simulation. The fitted model of aluminum is:

(1) $$BRDF=\frac{3.3\times 10^{-3}}{2.08\times 10^{-5}+{\left\|\beta -\beta_{0}\right\|}^{2}}$$

where ***β*** and ***β***_0_ are the projected vectors of unit vectors along specular reflection axis and observation direction respectively. In addition, we also set the workpiece as a perfect diffuser to evaluate the adaptability of our sensing method.

**Figure 8:**
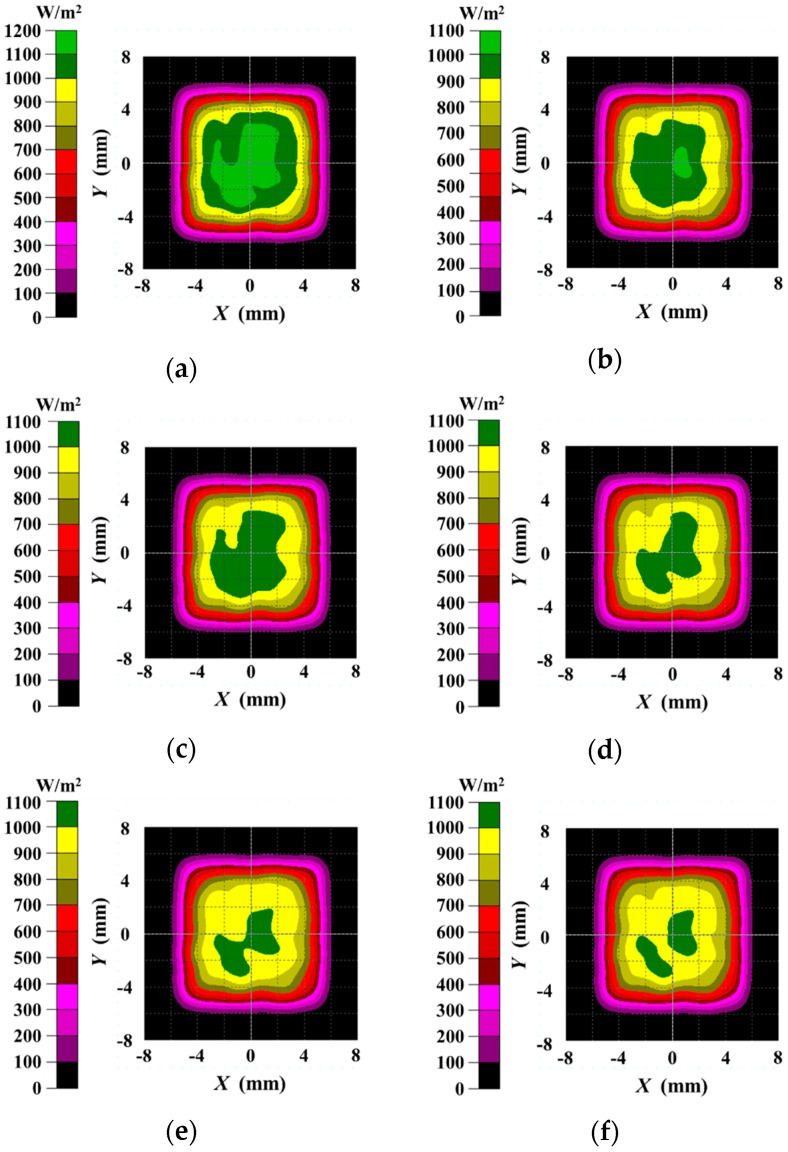
The luminous flux distribution in the region 0.5 mm above the top aperture. (**a**) The distance between the USLS and the workpiece surface is 30 mm and the reflection characteristic of the workpiece meets Equation (1); (**b**) The distance between the USLS and the workpiece surface is 30 mm and the workpiece is a perfect diffuser; (**c**) The distance between the USLS and the workpiece surface is 40 mm and the reflection characteristic of the workpiece meets Equation (1); (**d**) The distance between the USLS and the workpiece surface is 40 mm and the workpiece is a perfect diffuser; (**e**) The distance between the USLS and the workpiece surface is 50 mm and the reflection characteristic of the workpiece meets Equation (1); (**f**) The distance between the USLS and the workpiece surface is 50 mm and the workpiece is a perfect diffuser.

The simulation results are shown in Figure 8. The simulation results are similar whether the reflection characteristic of the workpiece meets Equation (1) or the workpiece is a perfect diffuser. In addition, the luminous flux distribution is nearly independent of the distance between the USLS and the workpiece surface when it varies from 30 mm to 50 mm. In the region −4 mm ≤ *X* ≤ 4 mm and −4 mm ≤ *Y* ≤ 4 mm (about 64% of the top aperture area), the luminous flux of each point is greater than 900 W/m^2^ and the distribution is quite uniform. The light emitted from the top aperture will be captured by the camera, which indicates that the grayscale distribution of the images would be uniform too. It can be seen that our proposed sensing method is effective to construct the uniform lighting condition, which can eliminate the grayscale inhomogeneity in the images due to the specular reflection effect.

**Figure 9:**
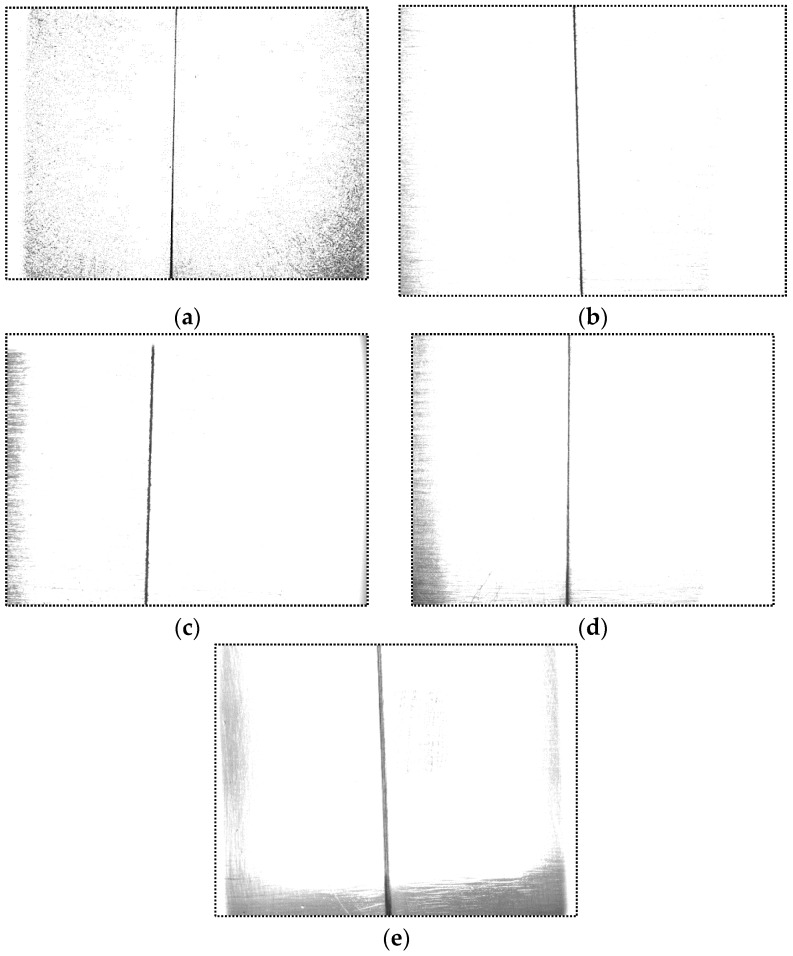
The original captured images of aluminum alloy with different surface roughness. (**a**) The surface roughness is about 12.5 μm; (**b**) The surface roughness is about 6.3 μm; (**c**) The surface roughness is about 3.2 μm; (**d**) The surface roughness is about 1.6 μm; (**e**) The surface roughness is about 0.8 μm.

Figure 9 shows the actual images captured by the camera using our method. The workpieces are all aluminum alloy whose surface roughness varies from 12.5 μm to 0.8 μm. Regardless of how the surface roughness varies, the grayscale in the base metal region is nearly saturated due to the strong specular reflection effect, while the grayscale in the groove region is nearly zero. We use the separation degree defined in Equation (2) to evaluate the grayscale differences between the base metal and the groove region:

(2) $$J=\frac{\left|\mu_{1}-\mu_{2}\right|}{\sqrt{\sigma_{1}^{2}+\sigma_{2}^{2}}}$$

where *μ*_1_ and *μ*_2_ are the average grayscales of the base metal and the groove region; *σ*_1_ and *σ*_2_ are the standard deviation of the grayscales in the base metal and the groove region. The calculation results of each image in Figure 9 is shown in Table 1. The separation degree is over 1.97 in our method. The grayscale differences between these two regions are greatly highlighted, which is helpful for an efficient and accurate detection and consequently improves the time complexity of the image processing procedure.

### 2.2. Image Processing Algorithm of Narrow Butt Detection

**Figure 10:**
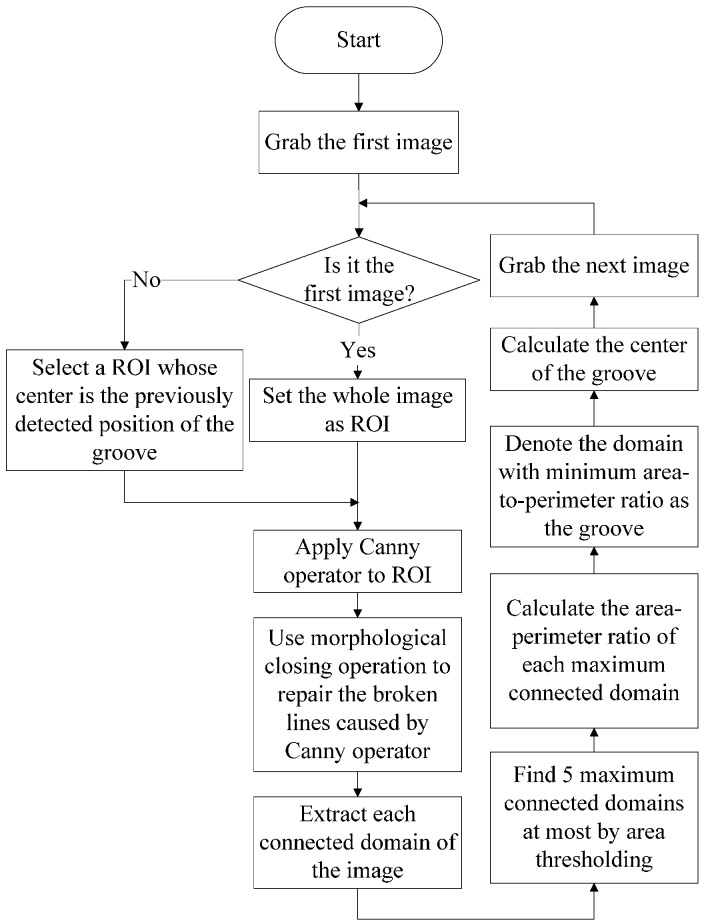
The image processing algorithm of narrow butt detection.

Based on the images captured by our sensing method, we propose a narrow butt detection algorithm as shown in Figure 10. The image processing algorithm was written in C++ and OpenCV library. Denote the current captured image as *I*_1_(*x*,*y*). The steps of the algorithm are as follows:

1. Region of Interest (ROI) setting. If the image *I*_1_(*x*,*y*) is the first one, set the whole image as ROI. Otherwise, the center of the ROI is located in the previously detected position of the groove; the width and the height of the selected ROI are all half of those of the whole image. Denote the ROI as *R*_1_(*x*,*y*).
2. Canny operation. Apply the Canny operator to *R*_1_(*x*,*y*) and a binary image *B*_1_(*x*,*y*) can be obtained containing the two-sided edges of the groove.
3. Morphological closing operation. The continuous groove region would become broken after the edge extraction process by the Canny operator. The morphological closing operation is used to repair the broken edges and fill the blank region between two edges of the groove as well. The image after morphological closing operation is denoted as *C*_1_(*x*,*y*).
4. Connected domain extraction and thresholding based on domain area. The flood-fill algorithm is used to extract each connected domain of the image *C*_1_(*x*,*y*). Then thresholding is used to eliminate the isolated connected domains with small areas. In this paper, at most five connected domains with maximum areas are left.
5. Find the domain with minimum area-to-perimeter ratio (APR) as the groove. In the groove region, the APR is about half of the groove width, which is usually small. In other regions with large areas, we can assume that these regions can be regarded as circles approximately. Therefore, the APRs of these regions are about a quarter of the width of themselves, which are usually much larger than half of the groove width. Calculate the APR of each connected domain and the minimum one indicates the groove region most probably.
6. Calculate the groove center. Scan each row or column of the groove region and we can finally obtain the trace of the groove center.

**Figure 11:**
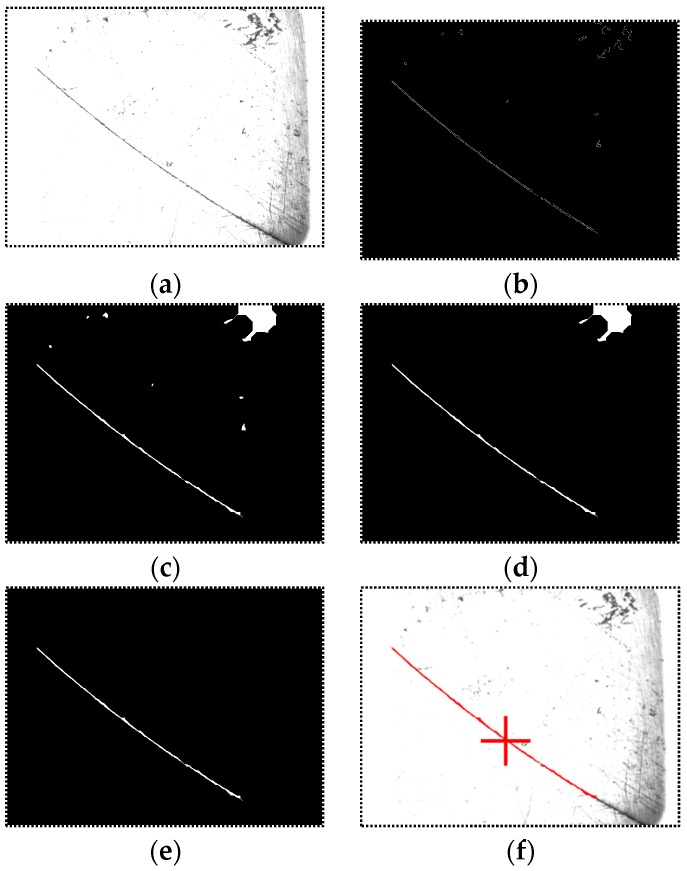
The processing result of each step of the narrow butt detection algorithm. (**a**) The original captured image; (**b**) The image after the Canny operator is applied; (**c**) The image after morphological closing operation; (**d**) The image after connected domain extraction and thresholding based on domain area; (**e**) The image after domain selection according to area-to-perimeter ratio; (**f**) The image after groove center calculation. The final detected results are marked as red curve.

Figure 11 shows the processing result of each step. In Figure 11d, there are two connected domains left after thresholding: the real groove region and a noise region on the upper right corner. The area of the noise region is larger than the groove so that it cannot be eliminated by thresholding based on domain area. After domain selection with minimum area-to-perimeter ratio, the real groove region can be found effectively.

## 3. Position and Pose Detection between the Welding Torch and the Workpiece Surface

The USLS we designed in Section 2 is used to generate the uniform lighting condition and detect the position of the groove in pixels, but it cannot detect the position and pose between the welding torch and the workpiece surface. As the transformation matrix between the camera and the torch coordinate systems can be determined by hand-eye calibration method, we only need to detect the equation of the workpiece surface in the camera coordinate system during welding. In this section, we will propose a double-line laser method to determine the equation of the workpiece surface.

**Figure 12:**
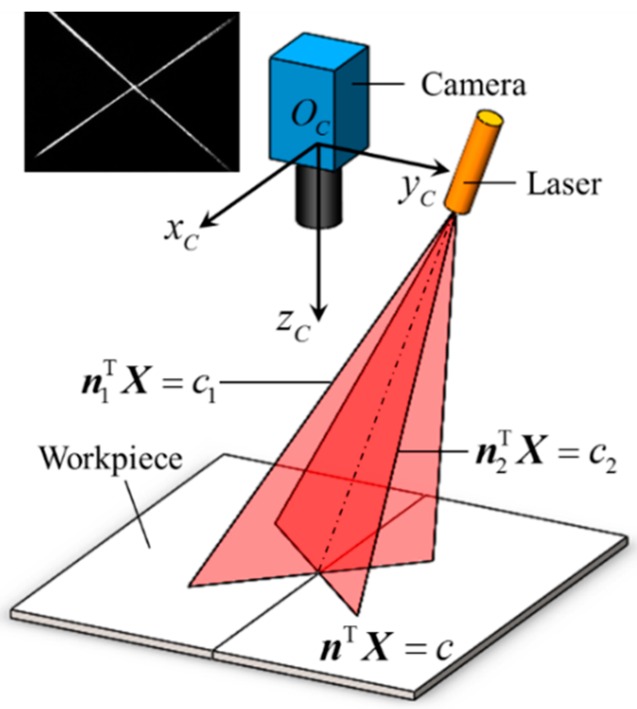
The principle of the double-line laser method.

Figure 12 shows the principle of the double-line laser method. A cross line laser stripe is projected onto the workpiece surface and the camera captures the grayscale images of the laser stripe. The intrinsic parameters of the camera and the equations of the laser light planes in the camera coordinate system {*C*} can be predetermined after calibration. These parameters can be used to calculate the 3D coordinate of each spot on the laser stripe. Therefore, the equation of the workpiece surface is determined in the camera coordinate system {*C*}. In the camera coordinate system {*C*}, suppose that the two light planes of the cross line laser are:

(3) $$n_{1}^{T}X=c_{1}$$

and:

(4) $$n_{2}^{T}X=c_{2}$$

respectively, where ***n***_1_ and ***n***_2_ are the unit normal vectors of the light planes, and *c*_1_ and *c*_2_ are the directed distances between the origin of {*C*} and the light planes. The parameters ***n***_1_, ***n***_2_, *c*_1_, and *c*_2_ can be predetermined using the Zhang’s calibration method [27].

Let us denote the pixel coordinates of the laser spot on each laser stripe as ***x***_1*i*_ and ***x***_2*j*_ respectively. According to the pinhole model of the camera, the 3D coordinate of ***x***_1*i*_ in {*C*} meets:

(5) $$X_{1i}=z_{1i}\cdot S(x_{1i})$$

where *z*_1*i*_ is an unknown parameter; ***S***(***x***_1*i*_) is a 3D vector related to the intrinsic parameters of the camera.

Combining Equations (3) and (5), we can determine the 3D coordinate of ***x***_1*i*_ by:

(6) $$X_{1i}=\frac{c_{1}}{n_{1}^{T}S(x_{1i})}\cdot S(x_{1i})$$

Similarly, the 3D coordinate of ***x***_2*j*_ can be expressed as:

(7) $$X_{2j}=\frac{c_{2}}{n_{2}^{T}S(x_{2j})}\cdot S(x_{2j})$$

Using Equations (6) and (7), we can obtain the 3D coordinates of all of the laser spots on the stripes. Denote them as ***G***_1_, ***G***_2_, …, and ***G****_N_* respectively. Assuming that the workpiece surface is a flat plane approximately, the Equation of the plane can be determined by the coordinates ***G***_1_, ***G***_2_, …, and ***G****_N_*.

Suppose that the Equation of the workpiece surface is:

(8) $$n^{T}X=c$$

where ***n*** is the unit normal vector of the surface, and *c* is the directed distance between the origin of {*C*} and the workpiece.

Since the laser spots are all located on the workpiece surface, there is:

(9) $$n^{T}G_{i}=c,\;i=1,2,\cdots ,N$$

The unknown parameter ***n*** and *c* can be solved by optimizating the following problem:

(10) $$\left\{ \begin{array}{l} \underset{n,c}{\min}f(n,c)=\underset{n,c}{\min}\frac{1}{N}\sum_{i=1}^{N}{\left|n^{T}G_{i}-c\right|}^{2}\\ s.t.\;\left\|n\right\|=1 \end{array}\right.$$

The Lagrange multiplier method is applied to solving Equation (10):

(11) $$g(n,c)=f(n,c)+\lambda (1-n^{T}n)=\frac{1}{N}\sum_{i=1}^{N}{\left|n^{T}G_{i}-c\right|}^{2}+\lambda (1-n^{T}n)$$

Calculate the partial derivatives of Equation (11) and set them to be zero:

(12) $$\left\{ \begin{array}{l} \frac{\partial g(n,c)}{\partial n}=2\left[\frac{1}{N}\sum_{i=1}^{N}\left(G_{i}G_{i}^{T}n-cG_{i}\right)-\lambda n\right]=0\\ \frac{\partial g(n,c)}{\partial c}=\frac{2}{N}\sum_{i=1}^{N}\left(c-G_{i}^{T}n\right)=0 \end{array}\right.$$

The solution to Equation (12) is

(13) $$\left\{ \begin{array}{l} \Omega \cdot n=\lambda \cdot n\\ c=n^{T}\bar{G} \end{array}\right.$$

where

(14) $$\left\{ \begin{array}{l} \Omega =\frac{1}{N}\sum_{i=1}^{N}(G_{i}-\bar{G}){\left(G_{i}-\bar{G}\right)}^{T}\\ \bar{G}=\frac{1}{N}\sum_{i=1}^{N}G_{i} \end{array}\right.$$

Combining Equations (10), (13) and (14), we can get:

(15) f(n,c)=λ

According to Equations (13) and (15), it can be inferred that the optimization problem in Equation (10) will be solved when ***n*** is the eigenvector of covariance matrix ***Ω*** corresponding to the minimum eigenvalue *λ*_min_. The unit normal vector ***n*** of the workpiece surface can be determined by Equation (13) and *c* can be also calculated by Equation (13) once ***n*** is determined. Considering ***Ω*** is a real symmetric matrix, the eigenvector ***n*** can be calculated by the Jacobi eigenvalue algorithm.

Let us denote the pixel coordinate of the groove center detected in Section 2 as ***γ***. Considering that the groove center is also located on the workpiece surface, the 3D coordinate of the groove center can be determined by:

(16) $$\Gamma =\frac{c}{n^{T}S(\gamma )}S(\gamma )$$

**Figure 13:**
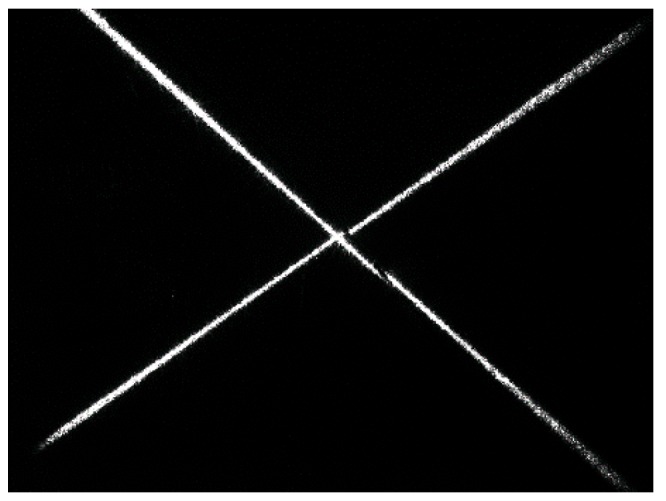
The original captured image of the cross line laser stripe.

**Figure 14:**
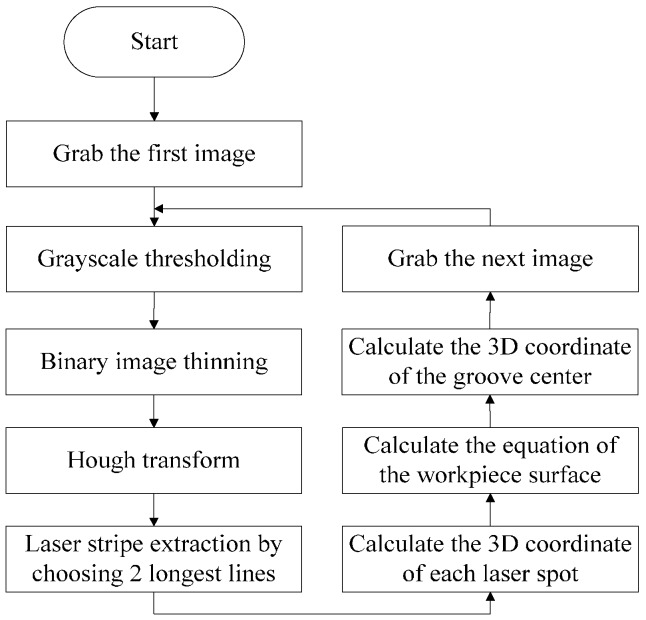
The image processing algorithm when laser light source is on.

Figure 13 shows the actual laser stripe image captured by the camera. We propose a corresponding processing algorithm to calculate the workpiece equation. The flow of the processing algorithm is shown in Figure 14. The laser stripes are extracted by Hough transformation first. Then the equation of the workpiece surface is determined by Equations (3) to (15), and the 3D coordinate of the groove center is determined by Equation (16).

## 4. Establishment of the Narrow Butt Detection Sensor

### 4.1. Configuration of the Narrow Butt Detection Sensor

**Figure 15:**
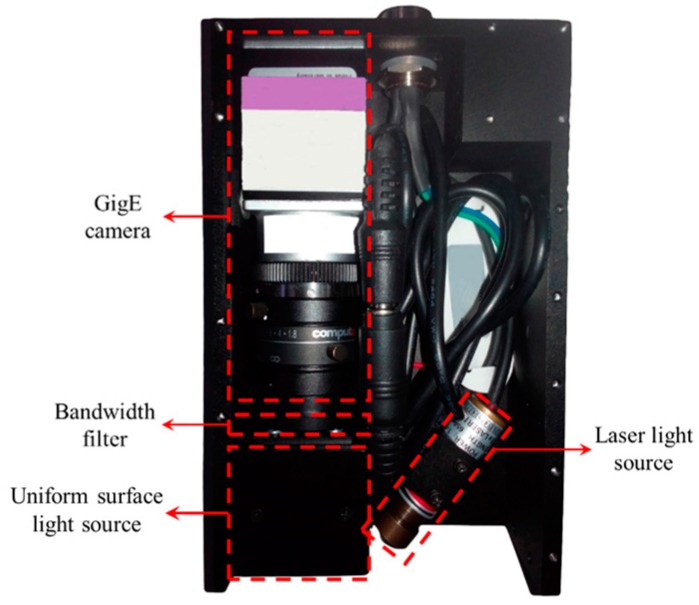
Configuration of the established narrow butt detection sensor.

Based on the research results above, we developed a narrow butt detection sensor as shown in Figure 15. It comprises a Gigabit Ethernet (GigE) camera, a USLS designed in Section 2, a laser light source (LLS), and a bandwidth filter. The GigE camera is an acA1600–60 gm (Basler, Ahrensburg, Germany) with 60 fps maximum frame rate and 1600 × 1200 pixels. The LLS emits cross line stripe with 20 mW power and 635 nm wavelength. The bandwidth filter is used to eliminate the interference from the arc and ambient light during welding. The central wavelength and Full Width at Half Maximum (FWHM) of the bandwidth filter are 635 nm and 10 nm, respectively. The USLS is designed to detect the pixel coordinates of the narrow butt, and the LLS is designed to detect the relative position and pose between the torch and the workpiece surface.

### 4.2. The “Shutter-Synchronized Dual Light Stroboscopic” Method

In order to obtain two kinds of visual information from the USLS and the LLS, we propose a “shutter-synchronize dual light stroboscopic” method to synchronize the camera and the two light sources. The USLS and LLS are switched on alternately and meanwhile, the camera is synchronized to capture images when each light source is on.

**Figure 16:**
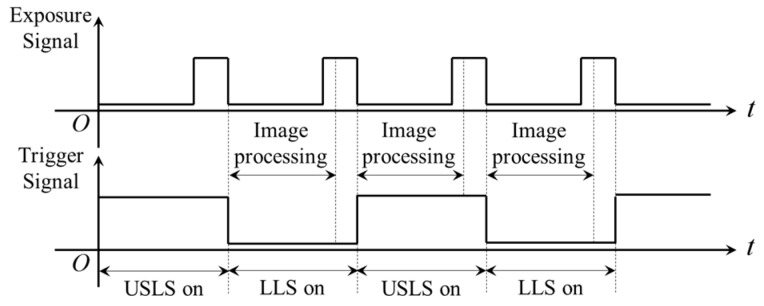
The correct method of generating the trigger signal.

**Figure 17:**
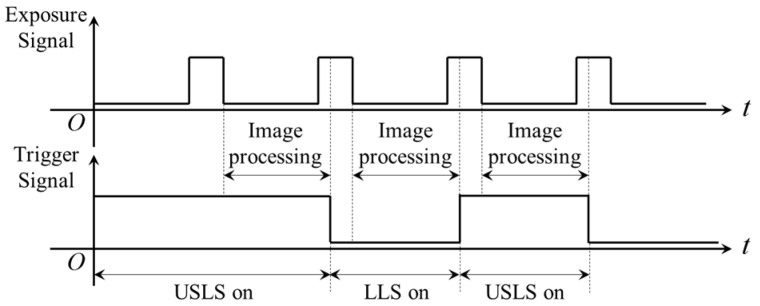
The incorrect way of generating the trigger signal.

In this paper, a TTL signal is used to trigger the USLS and the LLS. The trigger signal is generated from the digital output port of the camera. At the end of each exposure, the signal of the digital output port is inverted automatically by software as shown in Figure 16. Since the rising and falling edge of the trigger signal are both synchronized with the end of the exposure, the frequency of the trigger signal is exactly half of the frame rate of the camera. This signal is used to trigger the two light sources and they are switched on respectively during the high or low level period. It is noteworthy that the digital output port of the camera must be inverted at the immediate end of each exposure rather than after image processing. This is because the two light sources may be both switched on during one exposure as shown in Figure 17.

### 4.3. Images Captured in Different Distances and Angles

**Figure 18:**
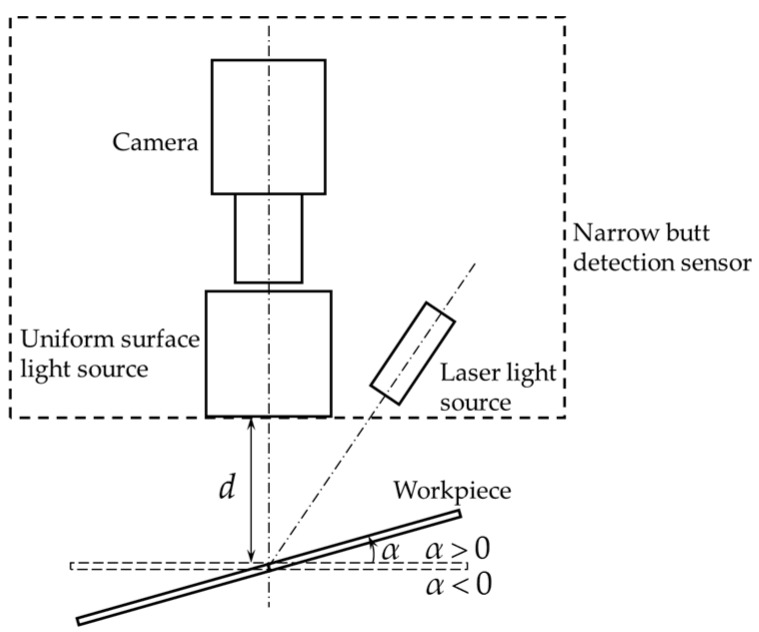
Relative positional relationship between the sensor and the workpiece.

Figure 18 shows the relative positional relationship between the sensor and the workpiece. We would study the images captured in different distances *d* and angles *α*.

**Figure 19:**
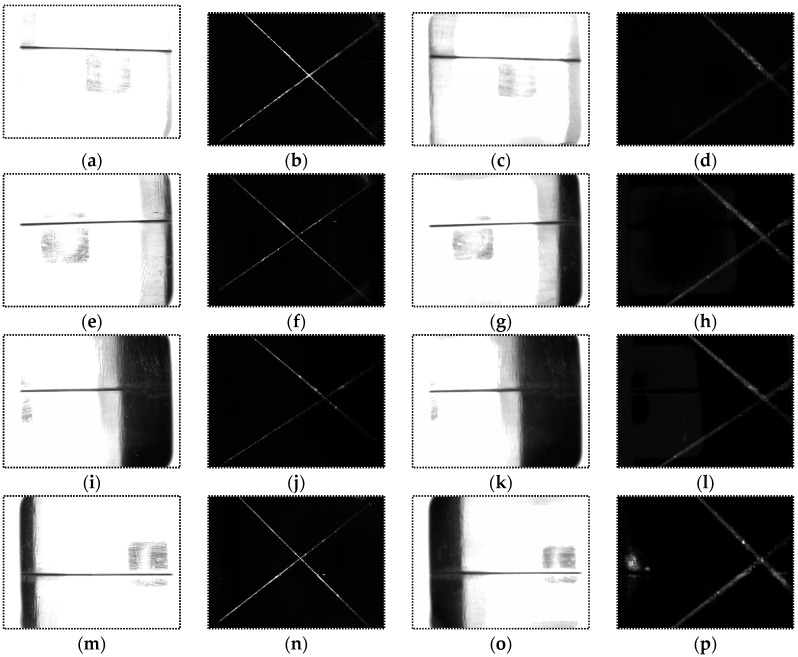

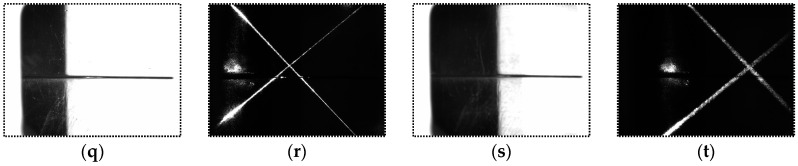
Images captured in different distances *d* and angles *α*. (**a**) USLS is on and *d* = 30 mm, *α* = 0°; (**b**) LLS is on and *d* = 30 mm, *α* = 0°; (**c**) USLS is on and *d* = 40 mm, *α* = 0°; (**d**) LLS is on and *d* = 40 mm, *α* = 0°; (**e**) USLS is on and *d* = 30 mm, *α* = 5°; (**f**) LLS is on and *d* = 30 mm, *α* = 5°; (**g**) USLS is on and *d* = 40 mm, *α* = 5°; (**h**) LLS is on and *d* = 40 mm, *α* = 5°; (**i**) USLS is on and *d* = 30 mm, *α* = 10°; (**j**) LLS is on and *d* = 30 mm, *α* = 10°; (**k**) USLS is on and *d* = 40 mm, *α* = 10°; (**l**) LLS is on and *d* = 40 mm, *α* = 10°; (**m**) USLS is on and *d* = 30 mm, *α* = −5°; (**n**) LLS is on and *d* = 30 mm, *α* = −5°; (**o**) USLS is on and *d* = 40 mm, *α* = −5°; (**p**) LLS is on and *d* = 40 mm, *α* = −5°; (**q**) USLS is on and *d* = 30 mm, *α* = −10°; (**r**) LLS is on and *d* = 30 mm, *α* = −10°; (**s**) USLS is on and *d* = 40 mm, *α* = −10°; (**t**) LLS is on and *d* = 40 mm, *α* = −10°.

**Figure 20:**
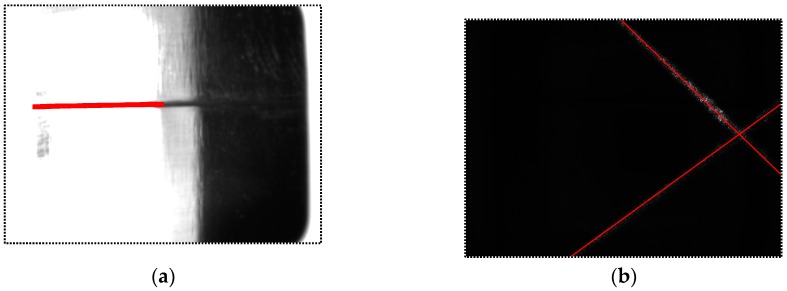
Image processing results. (**a**) Image processing results of Figure 19k; (**b**) Image processing results of Figure 19d.

Figure 19 shows the captured images when *d* = 30 mm, 40 mm and *α* = 0°, ±5°, ±10°. The angle *α* of the images in each row is 0°, 5°, 10°, −5°and −10° respectively; the distance *d* of the images in the 1st and 2nd column is 30 mm, while the distance *d* of the images in the 3rd and 4th column is 40 mm. When the USLS is on, the images become darker as the distance *d* and angle *α* increase. The intensity of the laser stripes in some images is low because of the specular reflection effects. All of these images are processed using our algorithm. Figure 20 shows the image processing results of Figure 19k,d, of which the imaging qualities are the worst in Figure 19. The processing results are marked as red curves in Figure 20. These results indicate that our detection method still works when the distance varies from 30 mm to 40 mm and the angle varies from −10° to 10°.

## 5. Experiments of Narrow Butt Detection and Discussions

### 5.1. Configuration of the Experiment Platform

**Figure 21:**
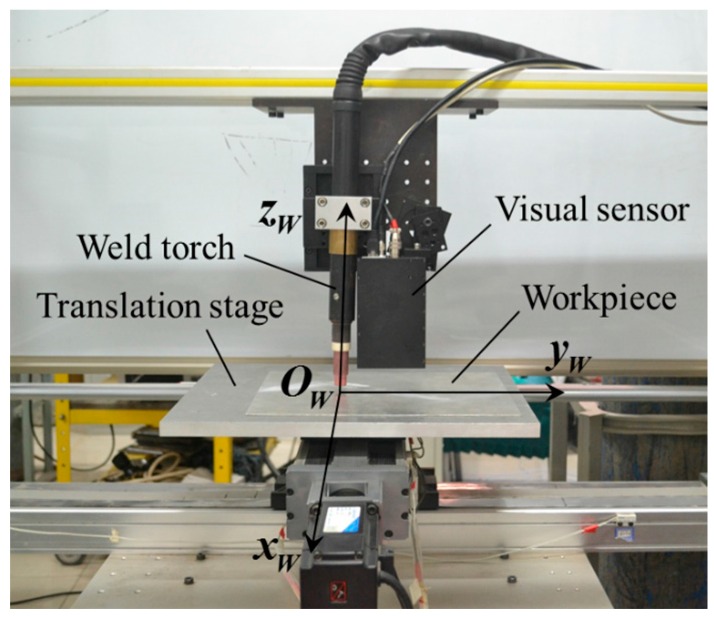
The established experiment platform for narrow butt detection.

The experiment platform was established in this paper as shown in Figure 21. The platform is comprised of a narrow butt detection sensor designed in Section 4, a translational stage, the GTAW welding equipment, and a computing unit. The sensor is fixed in front of the welding torch. The translation stage is controlled by ACR9000 motion controller, and the workpiece is placed on the terminal of the translation stage, so that the workpiece can move along X-axis and Y-axis with the translation stage. The velocity of each axis can be adjusted during the detection process by software. The axis of the welding torch is set to be perpendicular to both the X-axis and Y-axis of the translational stage. The computing unit is an industrial computer with 4 GB memory. The CPU of the computing unit is i7-3610 of which the clock frequency is 2.30 GHz.

An automatic path teaching operation is performed before welding using the narrow butt detection sensor. The workpiece will move along the Y-axis at a uniform speed. Meanwhile, the sensor detects the position of the narrow butt, and as a feedback, it is used to adjust the velocity of the X-axis in real time, keeping the narrow butt in the Field of View (FOV) of the camera during the whole teaching process. After that, the 3D groove path will be obtained, and the desired motion path of the workpiece during welding can be automatically generated offline.

During the welding process, the workpiece will move along the recorded path. However, the actual welding path may differ from the recorded one due to thermal deformation. As a result, the sensor has to detect the deviation between the recorded and actual path in real time during welding. Then the motion path of the workpiece will be adjusted automatically to correct the deviation.

**Figure 22:**
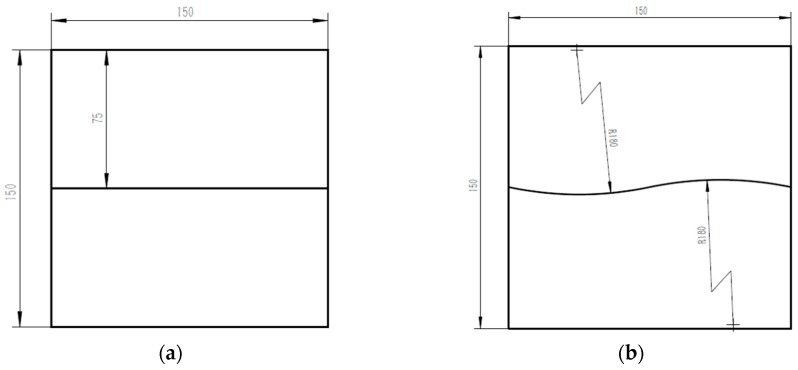
The CAD model of the workpiece samples. (**a**) A workpiece with the straight line path; (**b**) A workpiece with the S-curve path.

Figure 22 shows the workpiece samples used in our experiments. There are two samples: one with the straight path and the other with the S-curve path. They are both made of aluminum alloy with 5 mm thickness.

### 5.2. Motion Control System Design During Path Teaching

Denote the period of the trigger signal mentioned in Section 4.2 as *T_s_*. At the time *t* = *nT_s_*, the normalized pixel deviation between the FOV center and the groove center can be expressed as:

(17) $$e(n)=\frac{p_{i}(n)-p_{f}(n)}{M}$$

where *p*_i_(*n*) and *p*_f_(*n*) are the pixel coordinates of the FOV center and the groove center detected by our sensor, and *M* is the total row or column number of the image and it is equals to 2*p*_i_(*n*).

In this paper, we use the PID controller to adjust the velocity of the X-axis. Considering the velocity limit of the X-axis motor, the mathematical model of the controller in time domain is:

(18) $$v(n)=\min\left\{\max\left\{K_{P}e(n)+K_{I}\sum_{k=0}^{n}e(k)+K_{D}[e(n)-e(n-1)],-v_{\max}\right\},v_{\max}\right\}$$

where *v*_max_ is the maximum velocity of the X-axis, and *K*_P_, *K*_I_ and *K*_D_ are the gain parameters of the PID controller.

During the period between *t* = 0 and *t* = *nT*_s_, the displacement *s*(*n*) of the X-axis is:

(19) $$s(n)=\sum_{k=0}^{n}v(k)T_{s}$$

Applying Z-transform to Equation (19), there is:

(20) $$S(z)=\sum_{n=0}^{\infty }\left[\sum_{k=0}^{n}v(k)T_{s}\right]z^{-n}=\frac{T_{s}}{1-z^{-1}}V(z)$$

**Figure 23:**
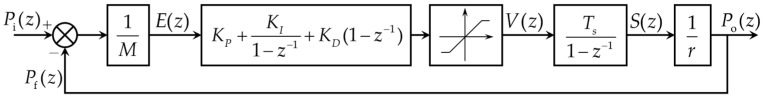
The block diagram of the control system.

The relationship between the displacement *s*(*n*) and the pixel coordinate of the groove center is approximately:

(21) $$p_{o}(n)=\frac{s(n)}{r}$$

where *r* is the resolution of the image in mm/pixel. In summary, the block diagram of the whole control system is shown in Figure 23.

**Figure 24:**
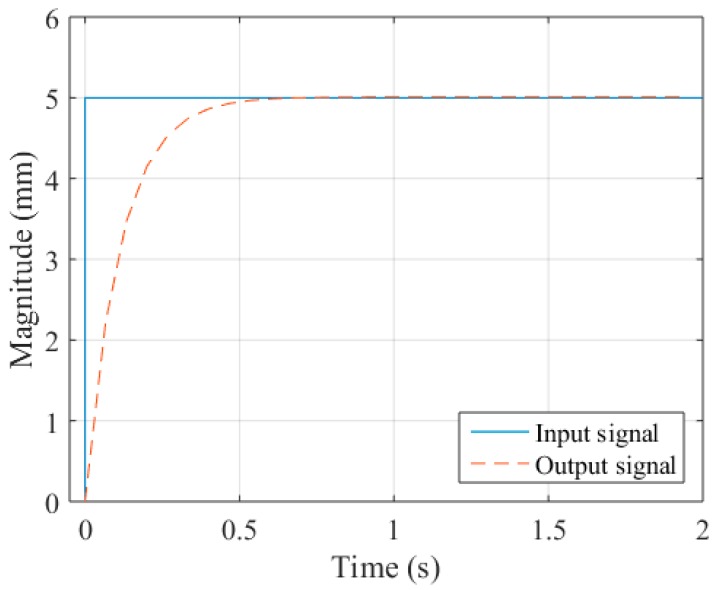
The step response of the proposed control system.

In this paper, the total row number of the image is 1200; the resolution *r* of the image is about 0.0125 mm/pixel; the frame rate of the camera is 30 fps, and the period of the trigger signal *T_s_* is 1/15 s; the maximum velocity *v*_max_ of the X-axis is 50 mm/s. The PID parameters *K*_P_, *K*_I_ and *K*_D_ are set to be 100, 0.1 and 0 respectively after careful tuning. Figure 24 shows the step response when the deviation between the groove and the center of FOV is 5 mm. The settling time *t*_s_ within ±5% error is about 0.333 s.

### 5.3. Path Teaching Results and Discussions

During the path teaching process, the velocity of the Y-axis is set to be 3 m/min. Experimental results show that the groove center is always located in the FOV of the camera during the whole teaching process. The time cost of image processing is respectively less than 10 ms and 5 ms when the USLS and the LLS is switched on. These results show that the proposed method is suitable for real-time detection.

Denote the point in the groove path as ***X****_i_*. At the time *t_i_*, we can calculate the coordinate ***X****_C_*_,*i*_ of the point ***X****_i_* in the camera coordinate system {*C*} using Equation (16). According to the coordinate transformation theory, the coordinate of the point ***X****_i_* at the time *t_i_* in the torch coordinate system {W} meets

(22) $$X_{W,i}=R_{CW}X_{C,i}+T_{CW}$$

where ***R***_CW_ and ***T***_CW_ are the rotation and translation matrices between {*C*} and {*W*}, which can be both predetermined by the common hand-eye calibration method.

Considering the displacements *l_x_*_,*i*_ and *l_y_*_,*i*_ of the translation stage at the time *t_i_*, the coordinate of the point ***X****_i_* in {*W*} at the initial time can be calculated by:

(23) $$X_{i}=X_{W,i}-\left[\begin{array}{c} l_{x,i}\\ l_{y,i}\\ 0 \end{array}\right]=R_{CW}X_{C,i}+T_{CW}-[\begin{array}{c} l_{x,i}\\ l_{y,i}\\ 0 \end{array}]\;\text{a}=1$$

**Figure 25:**
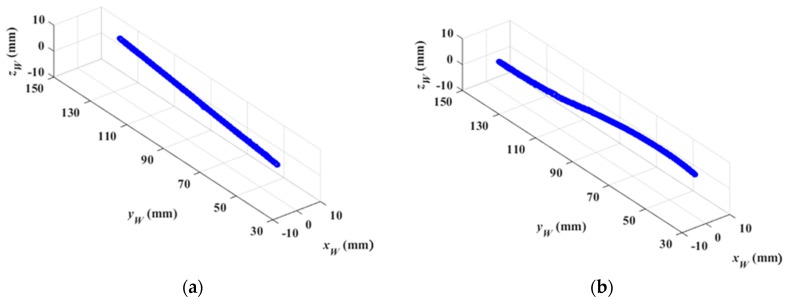
The 3D reconstruction of the groove path. (**a**) The straight line path; (**b**) The S-curve path.

Using Equation (23), we can calculate the coordinate of each point in the groove path at the initial time. Therefore, the 3D groove path can be reconstructed. The reconstructed results are shown in Figure 25. Compared with the theoretical CAD model in Figure 22, the maximum reconstruction errors are less than 0.45 mm and 0.56 mm.

**Figure 26:**
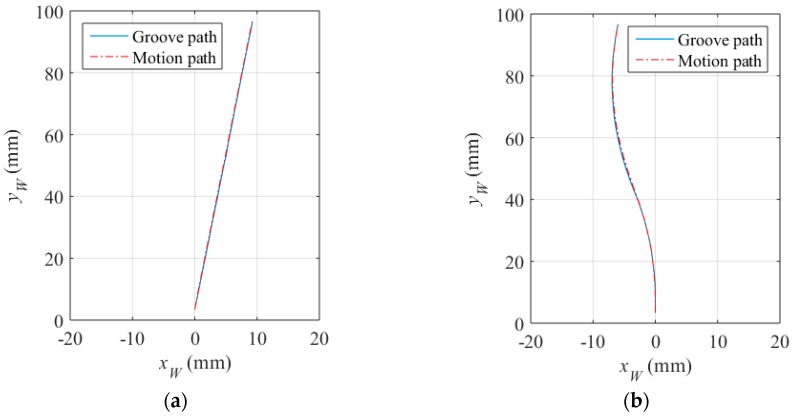
Comparison between the groove path detected and the motion path of the workpiece. (**a**) The straight line path; (**b**) The S-curve path.

Figure 26 shows the comparison between the groove path detected and the motion path of the workpiece during the teaching process. It can be seen that our proposed control method can perform an accurate tracking task during path teaching. The maximum deviations between the groove and the motion path are less than 0.11 mm and 0.17 mm.

### 5.4. Real-time Tracking Experiments during Welding and Discussions

**Figure 27:**
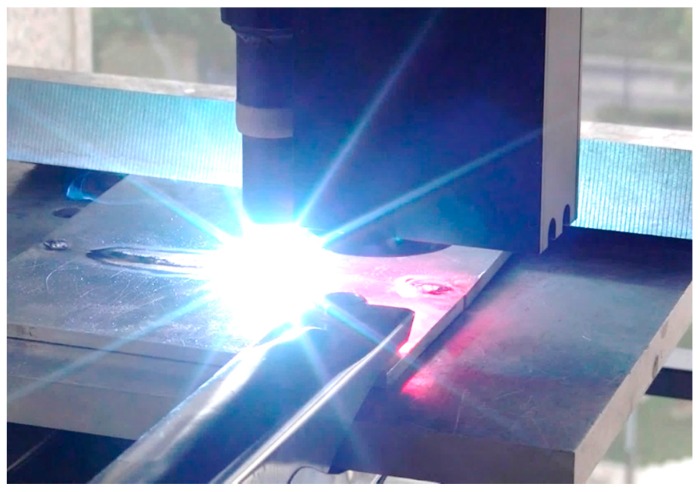
The welding scene in our experiments.

The motion path of the workpiece during welding can be automatically generated offline after path teaching. During the welding process, the actual groove path has to be detected in real time in order to correct the path deviations caused by thermal deformation. Figure 27 shows the GTAW welding scene in the experiments.

**Figure 28:**
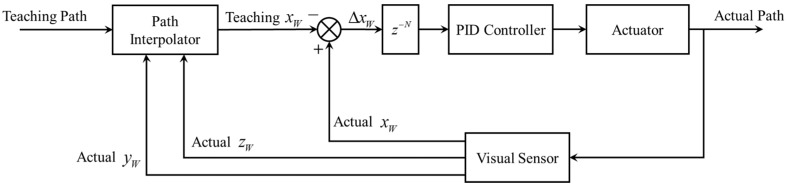
The motion control system during the welding process.

Figure 28 shows the motion control system during the welding process. The actual welding path is detected by our visual sensor and as a feedback, it is compared with the teaching path to calculate the deviations. Because the visual sensor is mounted in front of the welding region, the path deviations detected within a period of time must be stored in a First In First Out (FIFO) buffer, in order to correct the path deviation in the welding region, instead of the currently detected deviation in the FOV. The PID parameters of the control system in Figure 28 is set to be *K*_P_ = 100, *K*_I_ = 0.1 and *K*_D_ = 0 after careful tuning.

**Figure 29:**
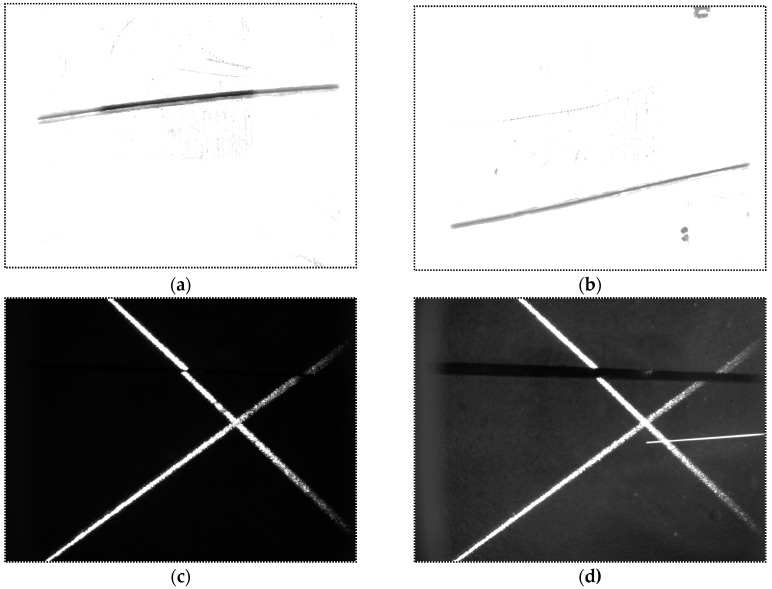
Images captured before and during welding. (**a**) The narrow butt image when the USLS is on before welding; (**b**) The narrow butt image when the USLS is on during welding; (**c**) The laser stripe image when the LLS is on before welding; (**d**) The laser stripe image when the LLS is on during welding.

In our experiments, the welding current and speed are set to be 160 A and 270 mm/min respectively. Figure 29 shows the images captured before and during welding. Comparing Figure 29b with Figure 29a, although the grayscales of the image during welding are larger overall, there are still significant grayscale differences between the groove and the base metal. This is mainly because the bandwidth filter in our sensor performs well in eliminating the interferences from the arc and ambient light. Furthermore, the area of the base metal is much larger than the groove, and the grayscale of the base metal is nearly saturated. As a result, most of the spatters would located in the base metal region, and they would rarely cover the groove, which has little influence on the imaging quality. In addition, the morphological closing operation of the algorithm in Figure 10 would repair the broken groove region even if the spatter covers it. Experiment results show that the image processing algorithm in Figure 10 performs well during the welding process and its time cost is less than 10 ms.

Comparing Figure 29d with Figure 29c, the grayscales of the laser stripes are still much greater than the background although the grayscales of the background increase during welding. The image may be interfered by the spatters, but it can be effectively eliminated by our proposed algorithm in Figure 14. Only two longest lines are regarded as the laser stripes in our proposed algorithm, so that the spatter in the image can be removed since it is much shorter than the laser stripes. The time cost of the image processing algorithm in Figure 14 is less than 5 ms in our experiments.

**Figure 30:**
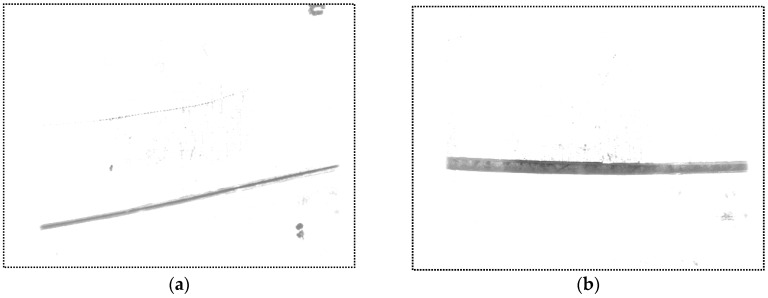
The narrow butt images captured during welding. (**a**) The image captured at the earlier time; (**b**) The image captured at the later time.

**Figure 31:**
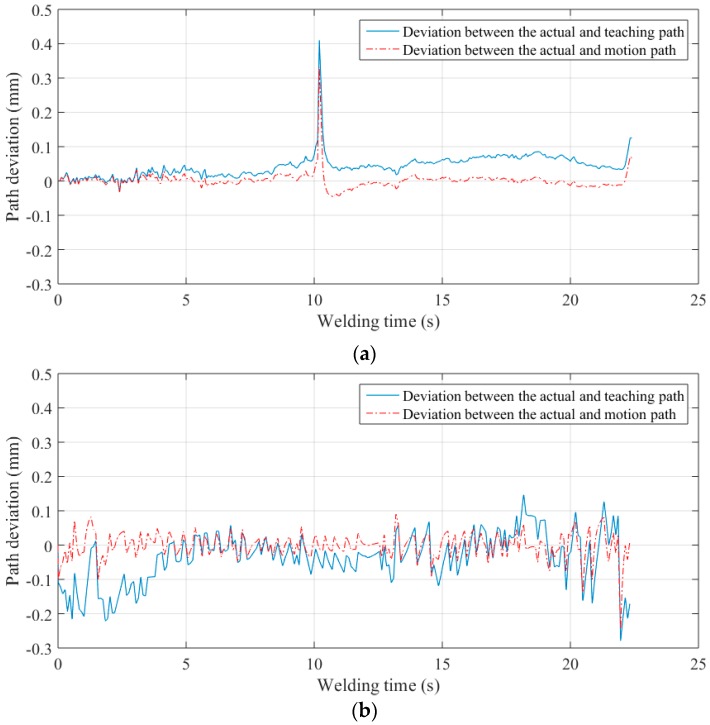
The path deviation during welding. (**a**) The path deviation during welding straight path; (**b**) The path deviation during welding S-curve path.

As the welding process goes on, the thermal deformation occurs as shown in Figure 30. The width of the groove increases gradually from about 0.2 mm to 0.8 mm. Figure 31 shows the deviation of the groove center between the actual and the teaching path. The maximum deviation of the groove center is about 0.41 mm. 

**Figure 32:**
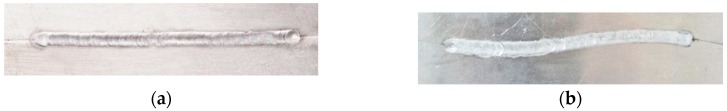
The welded workpiece with narrow butt. (**a**) The workpiece with the straight path; (**b**) The workpiece with the S-curve path.

These deviations have been corrected by our proposed control method in Figure 31, and the maximum deviation between the actual path and the motion path of the workpiece has decreased to about 0.33 mm during welding, as shown in Figure 31. Figure 32 shows the workpiece after welding.

**Figure 33:**
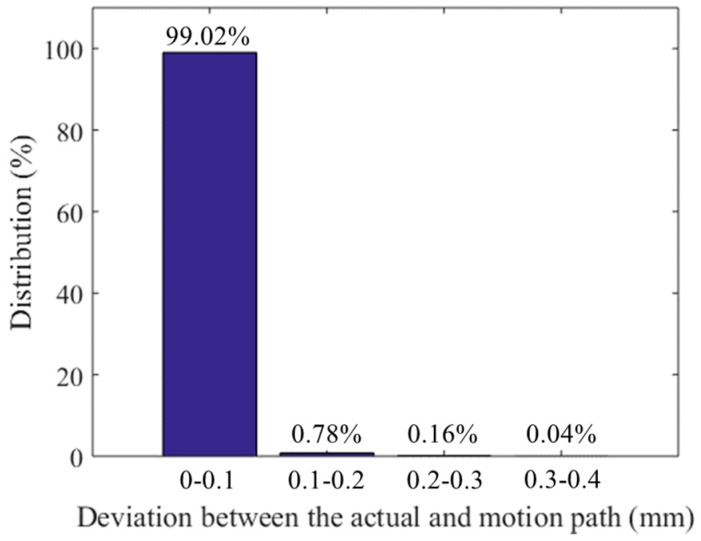
The distribution of path deviation between the actual path and the motion path of the workpiece during welding.

Besides the experiments mentioned above, 60 experiments are conducted when the welding current varies from 80 A to 200 A, the welding speed varies from 270 mm/min to 600 mm/min, and the materials are chosen are 6609 aluminum alloy, 2219 aluminum alloy, and stainless steel, respectively. Figure 33 shows the distribution of the path deviation between the actual path and the motion path of the workpiece during welding in these experiments. The deviation is usually less than 0.1 mm and the maximum one reaches less than 0.4 mm. All of these experiments show that our proposed method can resist the interference from the arc light, dust, and spatters. The proposed visual sensor is able to detect the accurate position of the narrow butt in the actual welding conditions, and the torch keeps aligned with the groove center during the whole welding process. It indicates that our proposed detection method is suitable in real time seam tracking fields during narrow butt welding of specular reflection workpiece.

## 6. Conclusions

This paper proposes a novel and precise visual detection method of narrow butt in specular workpiece welding. The designed uniform surface light source can create uniform lighting conditions on the workpiece surface and therefore, the grayscale distribution of the captured images is uniform, overcoming the adverse effects of specular reflection. Because of the uniform lighting condition and the reflection characteristic differences between the groove and the base metal, the grayscales of the groove are nearly zero while the grayscales of the base metal are almost saturated in the captured images. The SNR of the images are quite high and the grayscale differences between these two regions are greatly highlighted, which is beneficial to an accurate and efficient detection. In order to detect the relative position and pose between the torch and the workpiece, we propose a double-line laser method to calculate the equation of the workpiece surface. A “shutter-synchronized dual light stroboscopic” method is applied to acquiring two kinds of information when the uniform surface light source and the laser light source are both used. The uniform surface light source and laser light source are switched on alternately, and the camera is synchronized to capture the images when each light is on. In this way, we can obtain the 3D offsets of the groove center, and the relative pose between the torch and the workpiece surface nearly at the same time. Experimental results show that our proposed method is able to detect the narrow butt efficiently and effectively during the actual welding processes. The resolution of the images is 12.5 μm and the path deviation is less than 0.4 mm after control. The time cost of image processing is less than 10 ms and 5 ms, respectively, when the uniform surface light source and laser light source are switched on individually. These results show that the proposed method is suitable for automatic seam tracking during robotic welding processes. It can be applied to real-time narrow butt detection in specular reflection workpiece (such as aluminum alloy, titanium alloy, and stainless steel) welding. Further research will focus on its applications on complex 3D path welding and high-speed high energy beam welding.

## Figures and Tables

**Table 1:** The separation degree between the base metal and the groove region in Figure 9.

Figure 9	Separation Degree
a	3.740
b	4.354
c	4.657
d	3.581
e	1.973
